# The changing landscape of primary care: an analysis of payer-primary care integration

**DOI:** 10.1093/haschl/qxaf120

**Published:** 2025-06-11

**Authors:** Loren Adler, Samantha Crow, Matthew Fiedler, Richard Frank, Rahul Fernandez, Derek Lake, Robert Tyler Braun

**Affiliations:** Brookings Institution, Economic Studies, Washington, DC 20036, USA; Brookings Institution, Economic Studies, Washington, DC 20036, USA; Brookings Institution, Economic Studies, Washington, DC 20036, USA; Brookings Institution, Economic Studies, Washington, DC 20036, USA; Division of Health Policy and Economics, Weill Cornell Medical College, New York, NY 10065, USA; Cornell Health Policy Center (CHPC), Weill Cornell Medical College, New York, NY 10065, USA; Division of Health Policy and Economics, Weill Cornell Medical College, New York, NY 10065, USA; Cornell Health Policy Center (CHPC), Weill Cornell Medical College, New York, NY 10065, USA; Division of Health Policy and Economics, Weill Cornell Medical College, New York, NY 10065, USA; Cornell Health Policy Center (CHPC), Weill Cornell Medical College, New York, NY 10065, USA

**Keywords:** vertical integration, consolidation, primary care, Medicare Advantage, health insurance

## Abstract

**Introduction:**

Insurer ownership of primary care practices has expanded rapidly in recent years, but its magnitude, geographic distribution, and market drivers remain unclear.

**Methods:**

Using corporate filings, M&A databases, and insurer directories, we identify medical groups operated by UnitedHealth's Optum, Humana, Elevance, Aetna-CVS Health, and Cigna from 2016 to 2023 and calculate each payer's share of the Medicare primary care market—encompassing both Traditional Medicare and Medicare Advantage—nationally and by county. We then compare primary care market penetration by payers in counties above vs below the population-weighted median for hospital and insurer concentration, Medicare Advantage penetration, UnitedHealth's share of the Medicare Advantage and employer insurance markets, and hospital–physician integration.

**Results:**

Payer-operated practices account for 4.2% of the national primary care market by service volume in 2023, up from 0.78% in 2016. Optum, the largest payer-affiliated entity, held 2.71% nationally and over 35% in 3 large counties. The prevalence of payer-operated primary care was positively associated with Medicare Advantage penetration and negatively associated with concentrated hospital and employer-based insurance markets.

**Conclusion:**

Insurer control of primary care is expanding, concentrated in areas with robust Medicare Advantage enrolment and less concentrated hospital markets. Further research should examine its impact on care delivery, spending, and competition.

## Introduction

Health insurer ownership of physician practices has expanded significantly in recent years, drawing increased scrutiny from policymakers, researchers, and the public. In May 2024 testimony before the House of Representatives, UnitedHealth Group CEO Andrew Witty stated that their subsidiary Optum employs “just under 10 000” physicians—around 1% of all US physicians—and affiliates with another 80 000.^1^ But Optum is not alone in integrating with physician groups; other major insurers, including Humana, Elevance, and Aetna CVS Health, have also expanded their ownership of physician practices.

This trend has garnered widespread media attention, particularly regarding Optum's rapid expansion and its broader implications for the health care system.^2,3^ The Department of Justice has launched an antitrust investigation into UnitedHealth Group's increasing vertical integration, reflecting concerns about the competitive effects of payer ownership of physician practices.^4^ Policymakers and researchers have raised questions about how this integration affects patient care, health care costs, and market competition.

Despite reports of the growing prevalence and potentially far-reaching consequences, little is known about the extent of insurer–physician integration or the market conditions that drive it. In this paper, we provide the first estimates, to our knowledge, of the extent of insurer ownership of physician practices both nationally and at the county level.

Using Medicare data—covering both Traditional Medicare and Medicare Advantage (MA)—we quantify each payer's share of the primary care market from 2016 to 2023. We then examine the characteristics of markets where payer-primary care integration is most common, potentially offering insights into why insurers pursue these acquisitions. Our findings help establish a clearer picture of the evolving role of payers in physician practice ownership and suggest areas for further research.

## Study data and methods

### Data

#### Identifying payer-operated primary care groups

Building on work by Lake et al. (forthcoming) and Braun et al. (forthcoming), we created a dataset that identifies physician practices operated by Optum (part of UnitedHealth Group), Humana, Aetna CVS Health, Elevance, and Cigna, including the date of acquisition and the legal business name associated with the practice's taxpayer identification number (TIN) for the years 2016-2023. We consider a physician practice to be operated by a payer if it either is directly owned, part of a joint venture (eg, ChenMed), or has a market performance partnership with a payer (eg, John Muir Health and Optum^5^). We do not, however, classify an independent physician practice as payer-operated merely because it participates in an independent practice association (IPA) that affiliates with Optum or another insurer. We also exclude hospital-integrated payers such as Kaiser Permanente to isolate trends among insurers that are not fully integrated HMOs and non-profit insurers such as Blue Cross plans.

To construct this dataset, we first compiled lists of acquired practices using multiple data sources, including insurer websites, corporate registration databases such as HIPAASpace and OpenCorporates, US Securities and Exchange Commission filings, and merger and acquisition records from S&P Capital IQ and Irving Levin. These acquisitions were then linked to clinician billing data using the Medicare Data on Provider Practice and Specialty (MD-PPAS) file, which reports the National Provider Identifier (NPI) and the primary TINs physicians billed under each year along with the legal business name of each TIN. The MD-PPAS file, like the other non-public Centers for Medicare & Medicaid Services (CMS) data we discuss below, was accessed through CMS' Virtual Research Data Center. By cross-referencing the business names of acquired practices with MD-PPAS, we identified payer-operated TINs and documented when individual physicians began billing under them.

Because this process did not capture TINs that exclusively bill in MA—in which case we do not observe the TIN's legal business name—we undertook an additional process to identify such TINs operated by a payer. First, we compiled a list of all the organization NPIs billing under a known payer-operated TIN (identified in the prior steps) across both the Medicare Carrier (Part B) claims data and MA Encounter data. We then merged these data with information from the National Plan and Provider Enumeration System, which provides the name and title of each organization's designated authorized official, along with the organization's or medical group's name.^6^ By examining the authorized officials associated with known payer-operated groups, we identified additional organization NPIs and their corresponding TINs that were likely payer-operated based on continuity in leadership across payer-affiliated entities. These organization NPIs and TINs were then manually verified by cross-referencing the business name(s) used and the clinicians billing under each organization NPI and TIN. This manual process also identified some additional organization NPIs that used a different authorized official but shared a business name with known payer-operated groups.

Because MD-PPAS does not yet include data for 2023 and excludes providers who do not bill Traditional Medicare, we further supplemented our dataset with web-scraped information from insurer clinician directories and archived webpages. This final step allowed us to identify additional physicians practicing within payer-operated groups even in cases where Medicare claims data were unavailable.

#### Defining the primary care market

To identify primary care clinicians, we largely follow the classification methodology used in the Medicare Shared Savings Program (MSSP).^7^ Specifically, we define a clinician as primary care if they meet 2 criteria. First, they must be designated in Medicare data with a specialty of internal medicine, family practice, general practice, geriatric medicine, nurse practitioner (NP), physician assistant (PA), or certified clinical nurse specialist. Second, more than 70% of their total Medicare billings in a given year, measured by associated relative value units (RVUs), must be for primary care services as defined by the MSSP methodology. The main purpose of this second step is to allow us to distinguish NPs and PAs who provide primary care from those practicing in other specialties.

#### Market characteristics

We use 4 primary data sources to measure county-level market characteristics that may be associated with payer ownership of primary care practices. We focus on the county level, as MA markets are county-based, and using a consistent geographic unit facilitates comparisons across measures.

First, we measure hospital market concentration using data from the American Hospital Association (AHA). We calculate the Herfindahl–Hirschman Index (HHI) for each county, weighting by the total number of hospital beds within each health system (after pooling together hospitals that are part of the same health system, as identified by the AHA data).

Second, we assess insurance market concentration using insurer enrolment data from Clarivate Managed Market Surveyor (MMS), a widely used source in health care research. This dataset provides county-level enrolment figures for both the employer-sponsored insurance (ESI) market and MA. Using these data, we calculate HHIs to measure market concentration in each insurance sector. We also use the MMS dataset to determine UnitedHealthcare's share of local insurance markets.

Third, we measure MA penetration—defined as the share of eligible county residents enrolled in MA—using data published by the CMS.

Finally, to assess hospital-primary care vertical integration, we use data from IQVIA's SK&A Office-Based Physicians Database, which surveys physician practices to collect information on hospital affiliations, physician NPIs, and other practice characteristics. This dataset has been shown to include nearly all office-based physicians and is widely used in research on vertical integration.^8,9^ We identify primary care physicians in SK&A as those with a listed specialty of Family Practice, General Practice, or Internal Medicine. The SK&A dataset also indicates whether a practice is owned by or affiliated with a hospital or health system, which we use to calculate the share of primary care physicians in each county practicing within hospital-affiliated groups.

### Analytic approach

In this paper, we seek to estimate the prevalence of payer-operated medical groups in delivering primary care services and to better understand the features of local markets where this is more common.

#### Market shares

We quantify each payer's share of the primary care market at both the national and county levels for each year from 2016 to 2023. For each payer and year, we sum the RVUs associated with primary care services delivered by primary care clinicians (as defined above) who billed under payer-operated TINs across Traditional Medicare and MA. County locations are assigned based on the provider's ZIP code listed in the claim, which is mapped to the appropriate county using a crosswalk published by the US Department of Housing and Urban Development.^10^ The denominator for each county is the total RVUs associated with all primary care claims delivered by primary care clinicians in that county during the given year. Because Medicare claims data for 2023 were not yet available, we estimate these shares using ownership information from 2023 applied to service volumes from 2022.

#### Association with market characteristics

We also examine whether payer operation of primary care practices is associated with key county-level market characteristics, including:

- Medicare Advantage penetration
- UnitedHealthcare's share of the MA market
- Medicare Advantage carrier concentration
- Employer-sponsored insurance market concentration
- UnitedHealthcare's share of the employer health insurance market
- Hospital market concentration
- The share of primary care physicians employed by hospitals or health systems

To do so, we first calculate the population-weighted median of each market characteristic variable to allow comparisons between counties with above-median (“high”) vs below-median (“low”) levels of each variable. For each variable, stratified by “low” and “high” group of counties, we calculate the share of the population living in counties where payers operate <0.1% of the primary care market, between 0.1% and 5%, and over 5%. We also compute the overall payer share of the primary care market within each group. We repeat these analyses focusing exclusively on Optum's share of the primary care market.

Supplementary analysis in Appendix A shows estimates from county-level, cross-sectional multivariate ordinary least squares regressions to examine the relationship between the delivery of primary care services by payer-operated practices and these market characteristic variables.

### Limitations

This study has 2 primary limitations. First, while we are confident that our dataset captures most payer-operated practices delivering primary care services (among payers not primarily affiliated with a hospital), our ownership database is inevitably incomplete. We also intentionally exclude hospital-integrated payers such as Kaiser Permanente and non-profit payers. Additionally, we do not classify independent physician practices as payer-operated solely due to their participation in an independent practice association (IPA) that contracts with a payer for administrative support—we are unaware of a method to systematically identify such practices. We have heard anecdotal reports indicating that some payers have signed contracts of this type that have a long duration and that give the payer extensive control over practice operations. In such cases, this type of affiliation with a payer could function similarly to outright ownership.

Second, our method of identifying primary care clinicians may misclassify some clinicians. However, we conduct multiple sensitivity analyses to test the robustness of our classification approach.

## Results

### Market shares

Of the 381 463 primary care clinicians billing Medicare in 2023, 6.4% worked for a payer-operated practice (Table 1). Optum was the largest employer of both primary care physicians (4%) and advanced practice provider (2.8%).

**Table 1.** Breakdown of primary care clinicians by payer, 2023.

Category	Specialty	Total TM + MA clinicians	At least 1 MSSP PC code	MSSP PCP criteria	Optum PCPs	CVS/Aetna PCPs	Humana PCPs	Cigna PCPs	Elevance PCPs	Total PCPs at Payer-Operated Practices
Physicians	General practice	5774	5278	3892	190	3	189	8	60	450
	Family practice	109 755	105 485	84 409	3408	233	637	74	1181	5533
	Internal medicine	121 592	113 125	64 832	2538	144	716	38	979	4415
	Geriatric medicine	2017	1996	1428	34	9	7	2	37	89
	Total	***239 138***	***225 884***	***154 561***	***6170***	***389***	***1549***	***122***	***2257***	**10 487**
	Percent of physician PCPs				***4.0%***	***0.3%***	***1.0%***	***0.1%***	***1.5%***	**6.8%**
Advanced practice providers (APPs)	Nurse practitioner	263 666	243 689	165 549	4778	4823	659	38	1146	11 444
	Certified clinical nurse specialist	2656	2215	1316	40	1	1	—	5	47
	Physician assistant	131 973	119 441	60 037	1638	158	140	10	542	2488
	Total	***398 295***	***365 345***	***226 902***	***6456***	***4982***	***800***	***48***	***1693***	**13 979**
	Percent of APP PCPs				***2.8%***	***2.2%***	***0.4%***	***0.02%***	***0.7%***	**6.2%**
Total	Total	***637 433***	***591 229***	***381 463***	***12 626***	***5371***	***2349***	***170***	***3950***	**24 466**
	Percent of PCPs				***3.3%***	***1.4%***	***0.6%***	***0.04%***	***1.0%***	**6.4%**
All other clinicians		***1 302 559***	***726 395***							

Source: Authors' analysis of Traditional Medicare claims and Medicare Advantage Encounter data.

Abbreviations: MA, Medicare Advantage; MSSP, Medicare Shared Savings Program; PCP, Primary Care Provider; TM, Traditional Medicare.

**Figure 1.**
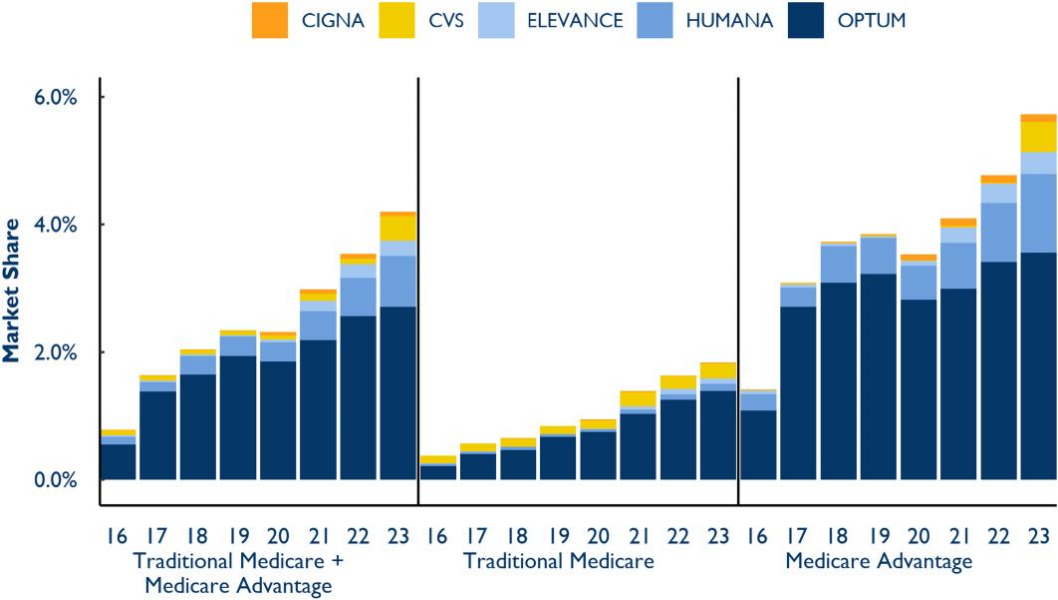
Primary care market share by payer, 2016-2023. Source: Authors' analysis of Traditional Medicare claims and Medicare Advantage Encounter data.

We also analyzed payers' penetration into primary care markets weighted by service intensity (measured by RVUs). The share of the national primary care market operated by payers grew from 0.78% in 2016 to 4.2% in 2023 (Figure 1). Optum drove much of that growth, with its share of the primary care market rising from 0.55% in 2016 to 2.71% in 2023. Notably, payer-operated practices delivered a substantially larger share of primary care services in MA (5.73%) than in Traditional Medicare (1.84%) in 2023.

**Figure 2.**
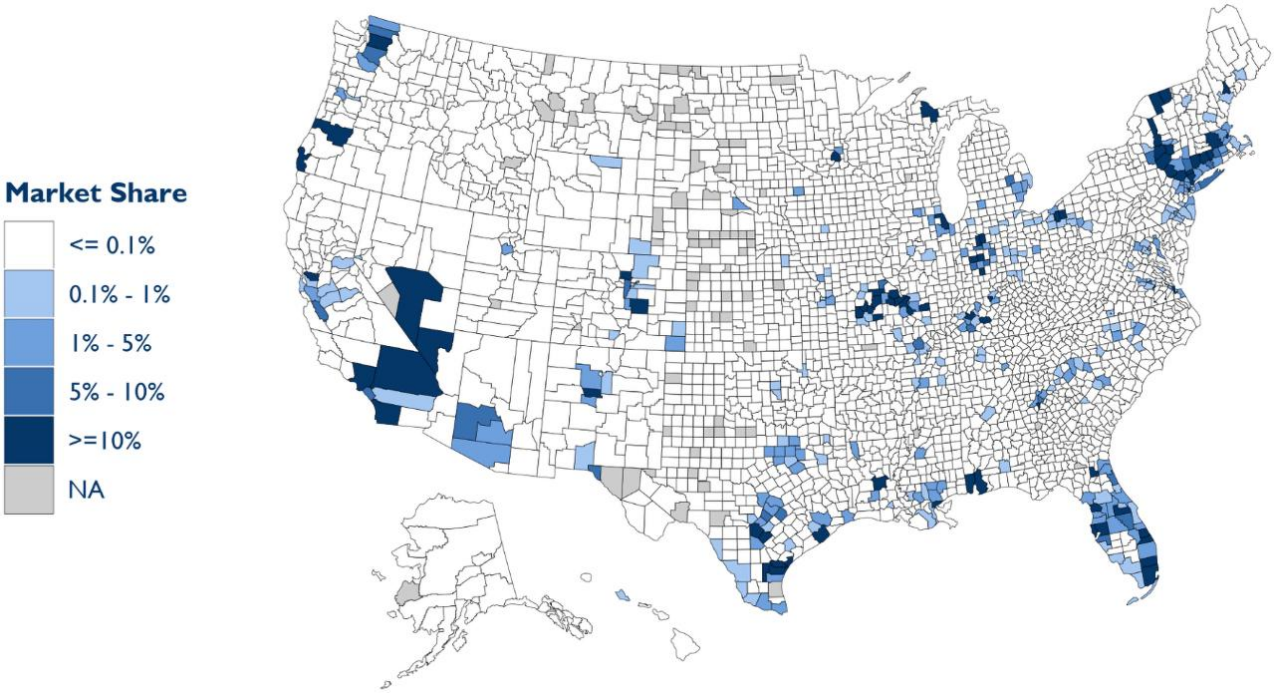
All payer primary care market share by county, 2023. Note: Gray counties have no primary care clinicians in our sample. Authors' analysis of Traditional Medicare claims and Medicare Advantage Encounter data.

These national figures obscure the substantial payer presence in certain counties (Figure 2). Among counties with some insurer presence in the primary care market, insurers operated practices responsible for 7.7% of local primary care services. And, in 2023, Optum controlled 44.9% of the primary care market in Snohomish County, WA, 40.1% in Contra Costa County, CA, and 35.8% in Clark County, NV. A full list of counties with a population >500 000 where payers operated at least 10% of the primary care market in 2023 is included in Appendix B, as well as an additional map depicting the location and local market penetration specific to Optum-operated practices. Overall, 15.1% of the US population resided in counties where payers controlled more than 10% of the primary care market in 2023, with 10.1% living in counties where Optum alone exceeded this threshold.

### Association with market characteristics

We examined how payer participation in primary care varied by market characteristics in 2022 (Table 2). In counties with less concentrated hospital markets (below-median hospital market HHI), 30.5% of the population lived in areas where payers operated <0.1% of the primary care market, compared with 75.3% in counties with more concentrated hospital markets. Similarly, payers controlled an average of 5.6% of the primary care market in counties with below-median hospital HHI, compared with only 1.5% in counties with above-median hospital HHI. We also find that payers operate a substantially greater share of the primary care market in counties with above-median MA penetration (5.5%) than in counties with below-median penetration (1.5%). Payer-operated primary care delivery is also notably more common in counties with below-median ESI market concentration (6.0% market share) than above-median concentration (1.2% market share). Table 2 presents similar breakdowns for other market characteristics, separately for all payers combined and for Optum specifically.

**Table 2.** Distribution of payer-operated primary care across market characteristics.

		Share of population in counties by market characteristics—2022			Payer primary care market share
		Payer share of primary care market			
		<0.1%	0.1%-5%	>5%	
All payer	Hospital market concentration				
	High HHI	75.3%	18.3%	6.5%	1.5%
	Low HHI	30.5%	38.1%	31.4%	5.6%
	Hospital-owned primary care market share				
	High share	61.6%	27.5%	10.9%	2.3%
	Low share	46.2%	27.6%	26.1%	4.9%
	ESI market concentration				
	High HHI	71.8%	21.3%	6.9%	1.2%
	Low HHI	35.7%	33.9%	30.3%	6.0%
	Medicare Advantage market concentration				
	High HHI	65.5%	25.3%	9.2%	2.7%
	Low HHI	42.3%	29.9%	27.8%	4.4%
	United MA market share				
	High share	54.4%	29.9%	15.6%	3.5%
	Low share	53.4%	25.2%	21.4%	3.6%
	United ESI market share				
	High share	46.7%	34.3%	19.1%	4.3%
	Low share	61.2%	20.9%	18.0%	2.7%
	Medicare Advantage penetration				
	High penetration	41.2%	29.0%	29.8%	5.5%
	Low penetration	66.6%	26.2%	7.2%	1.5%
	Distribution of population	53.9%	27.6%	18.5%	
	Number of counties	2777	186	97	
Optum	Hospital market concentration				
	High HHI	85.5%	10.8%	3.7%	1.1%
	Low HHI	47.3%	30.0%	22.6%	4.1%
	Hospital-owned primary care market share				
	High share	78.4%	14.2%	7.4%	1.8%
	Low share	55.9%	25.7%	18.4%	3.4%
	ESI market concentration				
	High HHI	86.9%	7.7%	5.4%	0.9%
	Low HHI	47.0%	32.4%	20.5%	4.3%
	Medicare Advantage market concentration				
	High HHI	76.5%	15.1%	8.4%	2.3%
	Low HHI	57.8%	24.8%	17.4%	2.8%
	United MA market share				
	High share	65.2%	23.6%	11.2%	2.9%
	Low share	69.1%	16.3%	14.6%	2.2%
	United ESI market share				
	High share	60.2%	29.3%	10.5%	3.0%
	Low share	74.2%	10.6%	15.3%	2.1%
	Medicare Advantage penetration				
	High penetration	53.8%	26.6%	19.5%	3.8%
	Low penetration	80.5%	13.3%	6.2%	1.2%
	Distribution of population	67.2%	20.0%	12.9%	
	Number of counties	2875	114	71	

“All Payer” represents the combined primary care market share of all payer-operated practices in a county. The population-weighted medians for each variable are as follows: hospital market concentration: 3296; hospital-owned primary care market share: 47%; ESI market concentration: 3380; Medicare Advantage market concentration: 2665; United MA market share: 25%; United ESI market share: 12.5%; Medicare Advantage penetration: 49%.

Source: Authors' analysis of Traditional Medicare claims and Medicare Advantage Encounter data, American Hospital Association Annual Survey Database, Clarivate Managed Market Surveyor, Centers for Medicare & Medicaid Services, IQVIA SK&A Office-Based Physicians Database.

Abbreviations: ESI, employer-sponsored insurance; HHI, Herfindahl–Hirschman Index; MA, Medicare Advantage.

## Discussion

Our findings illustrate the substantial and growing role that payers—particularly Optum—play in the US primary care market. Payer-operated practices accounted for 4.2% of Medicare primary care services in 2023, up from 0.78% in 2016. Optum, the largest payer-affiliated entity, controlled 2.71% of the national primary care market by service volume in 2023. The delivery of primary care services by payer-operated practices was especially pronounced in MA (5.73% nationally) relative to Traditional Medicare (1.84%). While much of the discussion surrounding payer–provider integration has focused on national trends, our results reveal significant geographic variation, with some counties seeing payer-controlled practices emerge as a dominant force in primary care.

Our analysis also highlights several key market characteristics associated with the local level of payer operation of primary care practices. Medicare Advantage penetration was strongly associated with payer market share—in 2022, payers operated 5.5% of the primary care market in counties with above-average MA penetration (over 49%) vs just 1.5% in counties with below-average MA penetration—suggesting that payers are more likely to integrate with primary care practices in counties where MA is more prevalent. Although the mechanisms behind this relationship require further exploration, prior research suggests that payer–provider vertical integration in MA increases diagnosis coding intensity, which raises government payments.^11^ Recent evidence also suggests that Optum may pursue such a strategy.^12^

Conversely, hospital market concentration was negatively associated with payer ownership of primary care practices. In counties with highly concentrated hospital markets, payers controlled a smaller share of the primary care sector. It is possible that hospital system concentration may constrain insurers' ability or desire to integrate with physician groups. Payer entry into primary care was also substantially less common in counties with highly concentrated ESI insurer markets. We observe similar patterns when focusing exclusively on Optum's presence in local primary care markets.

The rapid expansion of payer-owned primary care raises important policy considerations. On the one hand, vertical integration between insurers and physician practices could enhance care coordination, improve chronic disease management, and enable alternative payment models that shift incentives away from fee-for-service care. Greater control over referral patterns also allows for better steering to lower-cost settings such as ambulatory surgery centers (ASCs)—Optum, indeed, also acquired Surgical Care Affiliates, a large ASC chain—and may increase the insurer's bargaining power to negotiate lower prices with hospitals and specialists.^13^ And payer ownership of physician practices should reduce the inefficiencies associated with double marginalization, as the insurer and provider no longer set separate profit-maximizing markups. This integration increases the payer's incentive to reduce premiums because added enrollees now generate both provider-level and insurer-level profit margins.

However, increasing consolidation of primary care within payer-operated groups also raises concerns about competition and access. One concern is that payer-owned physician practices may be used to optimize risk adjustment coding, increasing government payments to their own MA plans without necessarily improving patient care.^12^ Vertical integration could also give insurers an advantage over competing health plans by steering patients toward their own services or making it harder for other insurers to contract with their physician groups. Additionally, payer acquisitions can directly reduce competition in local physician markets, potentially leading to higher prices or less choice for patients. These concerns have attracted growing regulatory scrutiny, with ongoing antitrust investigations into major payer–provider organizations and increasing calls for transparency in how these entities operate.

As payers continue to expand their presence in primary care, the implications for costs, competition, and patient outcomes remain uncertain. Future research should examine how this trend affects patient care, spending, and the broader health care market.

## Supplementary Material

qxaf120_Supplementary_Data
