# Supplementary material for: The changing landscape of primary care: an analysis of payer-primary care integration
Source: Health Aff Sch. 2025 Jun 11;3(7):qxaf120. doi: 10.1093/haschl/qxaf120 (PMC12223493; doi:10.1093/haschl/qxaf120)
Supplement: qxaf120_Supplementary_Data [file qxaf120_supplementary_data.zip › REVISED APPENDICES Payer-Primary Care (clean).pdf]

Appendix A. Regression Results

This appendix presents results from county-level, cross-sectional multivariate ordinary least squares regressions examining the relationship between the delivery of primary care services by payer-operated practices and the market characteristic variables described in the main text.

Methods

We divide each market characteristic variable into population-weighted terciles (i.e., such that roughly one-third of the U.S. population resides in each tercile). The dependent variable in our primary specification is the share of a county's primary care market operated by a payer in 2022. We also conduct a parallel analysis focusing specifically on Optum's share of the county primary care market.

Additionally, we assess the association between these market characteristics and a binary indicator for whether payers (or Optum specifically) operate at least 0.1% of the county's primary care market. We repeat this analysis with an alternative threshold of 1% for payer presence in the county primary care market.

All regressions are cross-sectional, conducted at the county level, and based on 2022 data on payer penetration into primary care. The population-weighted tercile cutoffs for each variable are as follows: Hospital HHI: 2,205 and 5,012; ESI Insurer HHI: 2,954 and 3,907; MA Insurer HHI: 2,351 and 3,062; United ESI Market Share: 9% and 17%; United MA Market Share: 20% and 35%; MA Penetration: 42% and 53%; Hospital/Primary Care VI: 35% and 57%.

Results

The primary specification indicates statistically significant multivariate associations between payer ownership of primary care practices in 2022 and several county-level market characteristics (Appendix Exhibits A1 and A2). One of the strongest predictors was Medicare Advantage (MA) penetration. In 2022, payers controlled a 2.94 percentage point larger share of the primary care market in counties with the highest tercile of MA penetration compared to in counties in the lowest tercile ($p<0.01$). Conversely, payer penetration into primary care was lower in counties where hospital markets and employer-sponsored insurance markets were more concentrated.

Similar results were observed in models where the outcome was payer market share exceeding 0.1% (chosen to represent a non-trivial level of payer penetration) and 1% (Appendix Exhibits A3 and A4).

Appendix Exhibit A1. Estimated association between payer share of primary care market and market characteristics (highest vs. lowest tercile), 2022

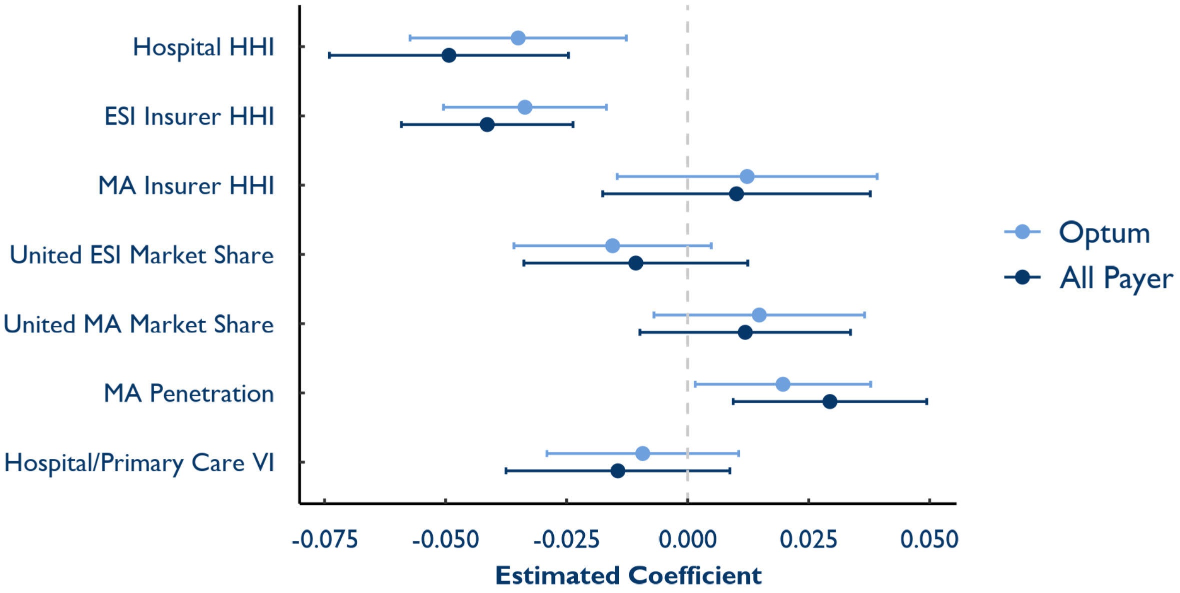

Note: These are the estimated coefficients on an indicator for the highest tercile of each variable, where the lowest tercile is the reference group. Bars around each point estimate reflect the 95% confidence interval. 'All Payer' represents the combined primary care market share of all payer-operated practices in a county.
Source: Authors' analysis of Traditional Medicare claims and Medicare Advantage Encounter data, American Hospital Association Annual Survey Database, Clarivate Managed Market Surveyor, Centers for Medicare & Medicaid Services, IQVIA SK&A Office-Based Physicians Database.

Appendix Exhibit A2. Full Regression Results: Payer Presence in Primary Care Markets and Market Characteristics

VARIABLES	(1) payershare	(2) optumshare	(3) payer_over01	(4) optum_over01	(5) payer_over1	(6) optum_over1
Tercile of Hospital HHI = 2	-0.0388***	-0.0259**	-0.279***	-0.294***	-0.251***	-0.196***
	(0.0136)	(0.0125)	(0.0611)	(0.0628)	(0.0682)	(0.0712)
Tercile of Hospital HHI = 3	-0.0493***	-0.0350***	-0.516***	-0.407***	-0.407***	-0.295***
	(0.0126)	(0.0114)	(0.0576)	(0.0556)	(0.0619)	(0.0623)
Tercile of ESI Insurer HHI = 2	-0.0232*	-0.0145	-0.0376	-0.0872	-0.0892	-0.00676
	(0.0121)	(0.0111)	(0.0520)	(0.0563)	(0.0620)	(0.0673)
Tercile of ESI Insurer HHI = 3	-0.0414***	-0.0336***	-0.206***	-0.310***	-0.281***	-0.207***
	(0.0090)	(0.0086)	(0.0590)	(0.0505)	(0.0553)	(0.0514)
Tercile of MA Insurer HHI = 2	0.00603	0.00442	-0.000127	0.00796	0.057	0.0307
	(0.0099)	(0.0092)	(0.0586)	(0.0557)	(0.0622)	(0.0677)
Tercile of MA Insurer HHI = 3	0.0101	0.0123	-0.0576	-0.0397	-0.0393	-0.0252
	(0.0141)	(0.0137)	(0.0585)	(0.0582)	(0.0632)	(0.0670)
Tercile of United ESI Market Share = 2	-0.0134	-0.0172**	0.00801	-0.109**	0.00909	-0.0911*
	(0.0083)	(0.0078)	(0.0485)	(0.0440)	(0.0498)	(0.0480)
Tercile of United ESI Market Share = 3	-0.0107	-0.0155	0.011	-0.0112	0.0145	-0.0537
	(0.0118)	(0.0104)	(0.0557)	(0.0609)	(0.0653)	(0.0718)
Tercile of United MA Market Share = 2	-0.00869	-0.0138*	0.0629	-0.013	-0.0509	-0.0918
	(0.0095)	(0.0082)	(0.0558)	(0.0547)	(0.0592)	(0.0609)
Tercile of United MA Market Share = 3	0.0119	0.0148	0.0999**	0.169***	0.0949*	0.185***
	(0.0111)	(0.0111)	(0.0508)	(0.0507)	(0.0541)	(0.0567)
Tercile of MA Penetration = 2	0.0101	0.00591	0.055	0.0842*	0.0727	0.0196
	(0.0081)	(0.0075)	(0.0514)	(0.0477)	(0.0516)	(0.0470)
Tercile of MA Penetration = 3	0.0294***	0.0197**	0.117**	0.130**	0.141**	0.0904
	(0.0102)	(0.0093)	(0.0526)	(0.0517)	(0.0554)	(0.0581)
Tercile of Hospital/Primary Care VI = 2	-0.0261**	-0.0156	0.00858	-0.119**	-0.0336	-0.0183
	(0.0133)	(0.0119)	(0.0560)	(0.0559)	(0.0637)	(0.0681)
Tercile of Hospital/Primary Care VI = 3	-0.0144	-0.00927	-0.0967*	-0.127**	-0.0845	-0.0544
	(0.0118)	(0.0101)	(0.0513)	(0.0510)	(0.0575)	(0.0606)
Constant	0.0888***	0.0670***	0.745***	0.708***	0.619***	0.469***
	(0.0215)	(0.0188)	(0.0897)	(0.0941)	(0.1080)	(0.1200)
Observations	2513	2513	2513	2513	2513	2513
R-squared	0.211	0.159	0.338	0.398	0.315	0.270

Robust standard errors in parentheses

*** $p<0.01$, ** $p<0.05$, * $p<0.1$

Notes: Models include robust standard errors and terciles are population-weighted.

Appendix Exhibit A3. Association between market characteristics (highest vs. lowest tercile) and payer presence (>0.1% market share) in county primary care markets, 2022

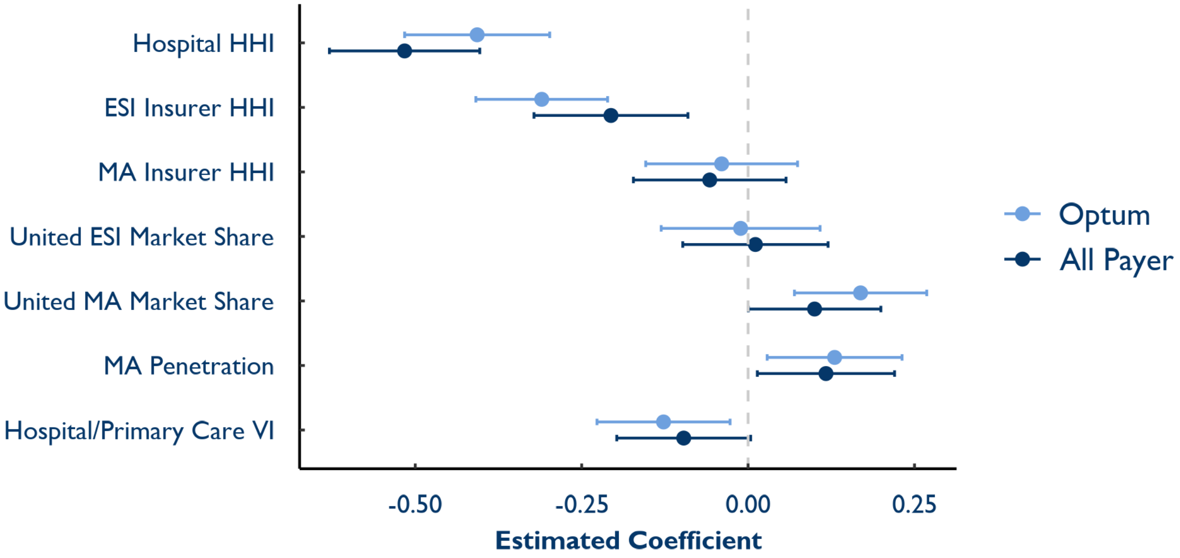

Note: These are the estimated coefficients on an indicator for the highest tercile of each variable, where the lowest tercile is the reference group. Bars around each point estimate reflect the 95% confidence interval. 'All Payer' represents the combined presence of all payer-operated practices in a county.
Source: Authors' analysis of Traditional Medicare claims and Medicare Advantage Encounter data, American Hospital Association Annual Survey Database, Clarivate Managed Market Surveyor, Centers for Medicare & Medicaid Services, IQVIA SK&A Office-Based Physicians Database.

Appendix Exhibit A4. Association between market characteristics (highest vs. lowest tercile) and payer presence (>1% market share) in county primary care markets, 2022

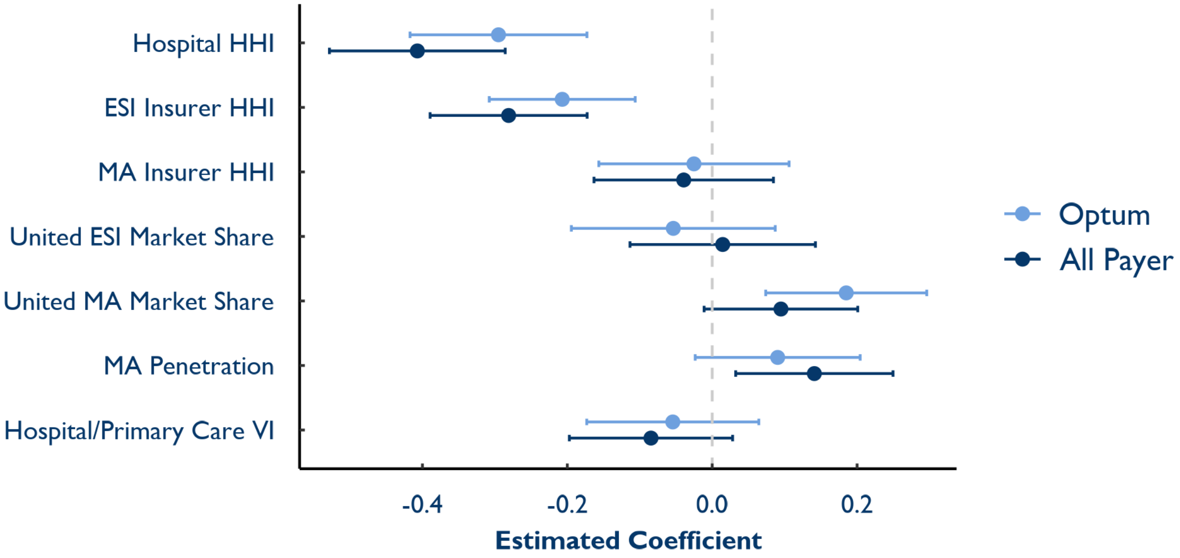

Note: These are the estimated coefficients on an indicator for the highest tercile of each variable, where the lowest tercile is the reference group. Bars around each point estimate reflect the 95% confidence interval. 'All Payer' represents the combined presence of all payer-operated practices in a county.
Source: Authors' analysis of Traditional Medicare claims and Medicare Advantage Encounter data, American Hospital Association Annual Survey Database, Clarivate Managed Market Surveyor, Centers for Medicare & Medicaid Services, IQVIA SK&A Office-Based Physicians Database.

Appendix B. Geographic Variation in Payer-Primary Care Integration

Appendix Exhibit B1. Payer Penetration into Large County Primary Care Markets, 2023

County	State	All Payer Market Share	Optum Market Share	Population
Snohomish County	WA	45%	45%	844,761
Contra Costa County	CA	40%	40%	1,155,025
Clark County	NV	38%	36%	2,336,573
Miami-Dade County	FL	36%	1%	2,686,867
Bexar County	TX	34%	31%	2,087,679
Hudson County	NJ	30%	30%	705,472
Hillsborough County	FL	24%	22%	1,535,564
Orange County	FL	22%	2%	1,471,416
El Paso County	CO	20%	20%	744,215
Westchester County	NY	20%	19%	990,817
St. Louis County	MO	19%	19%	987,059
Los Angeles County	CA	18%	13%	9,663,345
Broward County	FL	18%	1%	1,962,531
Suffolk County	MA	18%	18%	768,425
Bernalillo County	NM	18%	17%	671,586
Hennepin County	MN	18%	18%	1,258,713
San Diego County	CA	17%	0%	3,269,973
Worcester County	MA	15%	15%	866,866
Hartford County	CT	13%	13%	902,409
Pinellas County	FL	13%	11%	961,596
San Bernardino County	CA	13%	12%	2,195,611
Cook County	IL	12%	1%	5,087,072
Summit County	OH	11%	10%	535,733

Source: Authors' analysis of Traditional Medicare claims and Medicare Advantage Encounter data

Notes: This table illustrates the full list of counties with a population over 500,000 where payer-operated practices control at least 10% of the primary care market.

Appendix Exhibit B2. Optum Primary Care Market Share by County, 2023

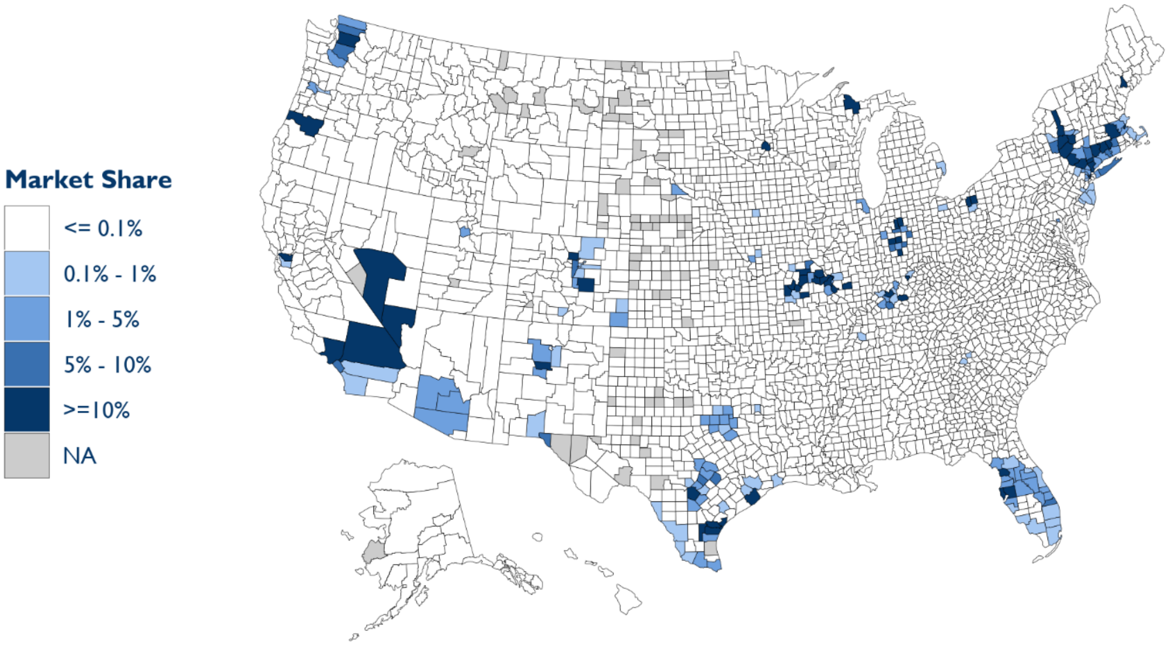

Note: Gray counties have no primary care provider in our sample.
Source: Authors' analysis of Traditional Medicare claims and Medicare Advantage Encounter data.
